# Remote magnetic actuation using a clinical scale system

**DOI:** 10.1371/journal.pone.0193546

**Published:** 2018-03-01

**Authors:** Jürgen Rahmer, Christian Stehning, Bernhard Gleich

**Affiliations:** 1 Philips GmbH Innovative Technologies, Research Laboratories, Hamburg, Germany; 2 Philips GmbH Market DACH, Hamburg, Germany; Massachusetts Institute of Technology, UNITED STATES

## Abstract

Remote magnetic manipulation is a powerful technique for controlling devices inside the human body. It enables actuation and locomotion of tethered and untethered objects without the need for a local power supply. In clinical applications, it is used for active steering of catheters in medical interventions such as cardiac ablation for arrhythmia treatment and for steering of camera pills in the gastro-intestinal tract for diagnostic video acquisition. For these applications, specialized clinical-scale field applicators have been developed, which are rather limited in terms of field strength and flexibility of field application. For a general-purpose field applicator, flexible field generation is required at high field strengths as well as high field gradients to enable the generation of both torques and forces on magnetic devices. To date, this requirement has only been met by small-scale experimental systems. We have built a highly versatile clinical-scale field applicator that enables the generation of strong magnetic fields as well as strong field gradients over a large workspace. We demonstrate the capabilities of this coil-based system by remote steering of magnetic drills through gel and tissue samples with high torques on well-defined curved trajectories. We also give initial proof that, when equipped with high frequency transmit-receive coils, the machine is capable of real-time magnetic particle imaging while retaining a clinical-scale bore size. Our findings open the door for image-guided radiation-free remote magnetic control of devices at the clinical scale, which may be useful in minimally invasive diagnostic and therapeutic medical interventions.

## Introduction

For controlling small devices inside the human body, many actuation and propulsion methods have been suggested, deriving their energy from various sources such as on-board batteries, chemical and physical energy harvesting, or wireless energy transfer [1]. Due to the limited space available in micro-devices, wireless energy transfer from the outside using magnetic, electric, electromagnetic (radio-frequency), optical, or vibrational energy is most widely used [1,2]. Magnetic energy transfer by remote magnetic actuation is one of the most simple and mature approaches [3,4]. It is capable of delivering forces and torques for propulsion and steering without the need for a local device-bound power source [5]. Remote magnetic control is used routinely to steer catheters in cardiac ablation procedures [6,7], but it also has high potential for controlling untethered devices like camera pills in the gastro-intestinal tract [8–10] or drills operating in tissue [11] or in the vascular network [12–14]. Magnetic fields are furthermore applied for propulsion and steering of micro-robots in potential future diagnostic and therapeutic applications, such as local sensing [1], local drug delivery [15,16] or remotely controlled minimally invasive surgery [17]. For the generation of both torques and forces, a field generator should be able to produce strong homogeneous fields and strong field gradients, respectively [18,19]. A number of powerful magnetic field generators with many degrees of freedom have been built with small workspaces for experimental or pre-clinical use [12,14,20,21]. For medical applications, it is necessary that the magnetic field generator can accommodate a patient and that the workspace in which the required magnetic field configurations can be applied is sufficiently large. Commercially available catheter steering systems (Niobe system, Stereotaxis, St. Louis, USA) fulfil these requirements [6] and are used in conjunction with X-ray systems for catheter localization. In that case, the desired field configurations are created by mechanical alignment of permanent magnets and are limited to homogeneous fields of rather low magnitude (about 80 mTμ_0_^−1^). These are sufficient to produce torques on the catheter tip to steer it into a desired direction, but lack the versatility and speed needed for controlling untethered devices. A coil-based field applicator demonstrated by Siemens and Olympus for steering camera pills for endoscopy of the gastro-intestinal tract [10] enables more degrees of freedom. Its field strength (up to 100 mTμ_0_^−1^) and gradient (achieving forces up to 1 mN) are high enough for operating a camera pill inside the stomach; other applications using this field applicator have not been reported. Other clinical-scale coil-based electro-magnetic actuation systems have been presented with homogeneous fields up to 140 mTμ_0_^−1^ and gradient fields up to 0.7 Tm^-1^μ_0_^−1^ [22–24]. While these systems rely on X-ray imaging for guidance and thus expose the patient to ionizing radiation, commercial magnetic resonance imaging (MRI) systems can be used for remote magnetic manipulation and offer the possibility of device localization by interleaving imaging with the manipulation sequences (magnetic resonance navigation–MRN) [25–27]. Although promising results have been demonstrated, MRI machines are not optimal for magnetic actuation. The forces on magnetic objects are limited due to the rather low strength of the field gradient (up to 60 mTm^-1^μ_0_^−1^ in standard clinical systems) and the field direction cannot be changed due to the static homogeneous main field of more than one tesla, which is typically generated using a superconducting coil that cannot be switched easily [28]. Here we present initial results on magnetic actuation using a newly developed clinical-scale field applicator that is large enough to accommodate a patient and has a workspace of more than 20 cm in diameter [29,30]. Using oil-cooled coils with soft-magnetic cores, it can generate dynamic homogeneous fields of up to 100 and 400 mTμ_0_^−1^ within the workspace in horizontal and vertical direction, respectively. Rotating uniform fields with frequencies of several hertz are applied to drill helical objects on curved 3D trajectories through gel or tissue. The coils can also generate permanent field gradients up to 2.0 Tm^-1^μ_0_^−1^ in vertical direction, which enables spatially selective actuation of identical helical micro-machines with a spatial resolution of a few millimeters [31]. The field applicator is part of a magnetic particle imaging (MPI) system and initial imaging results are presented. The system thus has the potential for closed-loop image-guided steering of devices without the need for ionizing radiation [32,33].

## Materials and methods

### Field generator and field control

**Fig 1:**
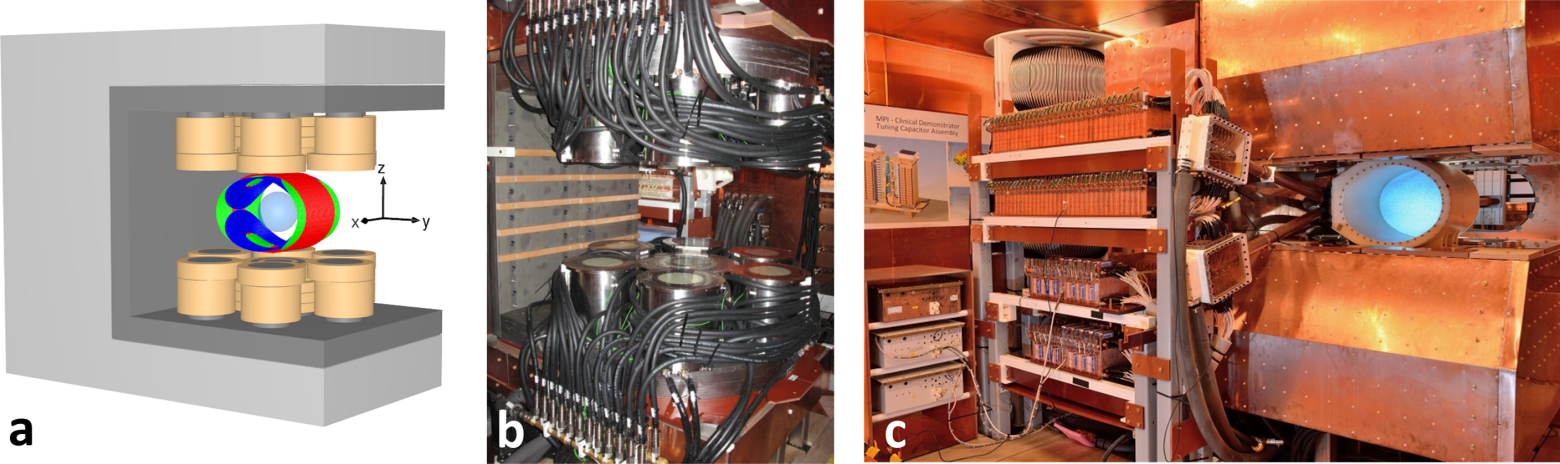
Clinical scale field applicator. (a) Schematic drawing of stacked coils (copper color), iron core (dark gray), and steel yoke (light gray) for low-frequency field generation (< 100 Hz) and flux guidance. A total of 18 coil stacks can be addressed individually by 18 amplifiers. Both at the top and bottom, six outer stacks, a cylindrical stack consisting of two segments, and a central inner stack are mounted. The elliptical coils (red, blue, green) are used for high-frequency imaging signal generation and reception and have not been used in the manipulation experiments. The transparent blue sphere indicates the 20 cm workspace. (b) Photograph of low-frequency field applicators. Gray hoses supply cooling oil, while black cables are the current connectors. (c) Final assembly of clinical MPI demonstrator. The low-frequency field generator has been completely shielded to avoid contact between the high-frequency excitation field and materials with a non-linear response. Left of the field generator, capacitors and toroidal coils are visible. Together with the drive coil (white coil with blue light), these form the resonant three orthogonal transmit/receive channels operating at approx. 150 kHz.

**Fig 2:**
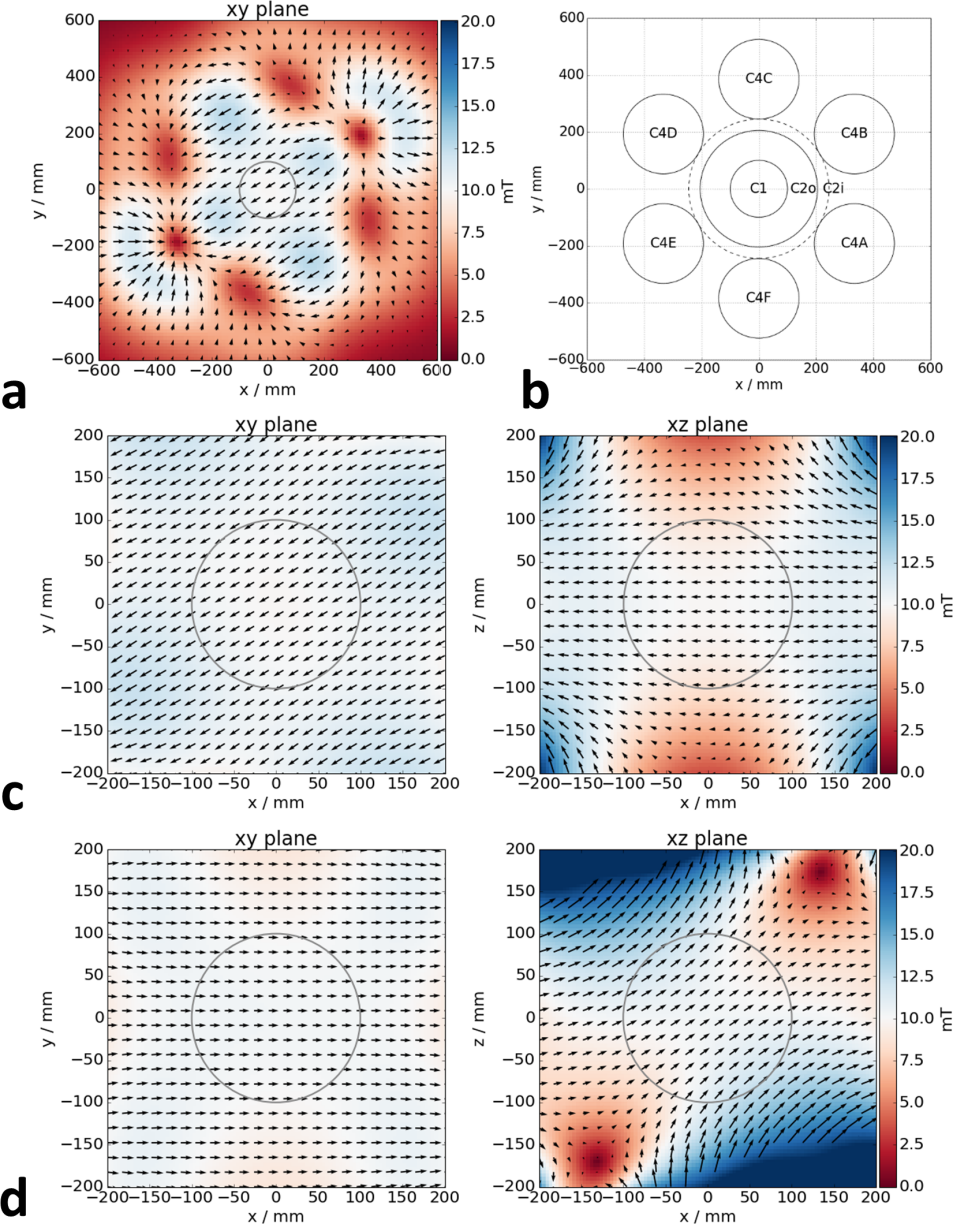
Field distribution. For assessment of the field homogeneity over the spherical workspace with a diameter of 20 cm, a simple linear field calculation was performed for a desired field amplitude of 10 mT/μ_0_. (a) Overview of field configuration in central xy plane for field aligned in the plane. The workspace is indicated by the gray circle. (b) Positions and labels of individual coil stacks. (c) Snapshot of field configuration for field rotation in the xy plane. The field strength and orientation is plotted for the central xy (left) and xz plane (right), respectively. The full rotation sequence can be found in S1 video. (d) Snapshot for field rotation in the xz plane. The full rotation sequence can be found in S2 video.

The clinical-scale field generator consists of 18 stacks of copper coils enclosed in 16 steel cylinders as shown in Fig 1. A laterally open design of the field generator has been chosen to enable good access to a patient. All coil stacks have cylindrical soft-magnetic iron-silicon cores for amplifying the field flux. The stacks are mounted on a non-magnetic steel gantry on which further soft-magnetic material is attached for guiding the flux along the inner side of the gantry. Electrically, coils within a stack are combined in series and each stack is connected to an MRI gradient amplifier for current supply (IECO, Finland). Maximum currents between 110 and 140 A were applied per stack. For cooling, the coil stacks are connected to a cooling circuit filled with transformer oil (Mobiltherm 594, ExxonMobil, USA) with a total cooling capacity of 100 kW (Schwämmle, Germany). The positions and labels of the coils stacks are shown in Fig 2B. The twelve outer steel cylinders (C4A to C4F), six at the top and six at the bottom, as well as the center cylinders (C1) at the top and at the bottom, all contain a single coil stack. In the two ring-shaped cylinders placed around the center coils, the stacks are segmented vertically into two parts (C2o and C2i), each driven by an individual amplifier.

For the generation of specific field configurations inside the spherical workspace, a field-to-current conversion model is necessary to determine the required currents on the 18 amplifier channels. Ideally, this model should also account for non-linearities resulting from the presence of partially saturated soft-magnetic material. This non-linear model has not been developed yet and therefore a simple linear model has been implemented that is based on the symmetry of the setup. For a desired homogeneous field **H**, the current vector **I**(**H**) containing the currents on all channels was calculated according to:

$$I\left(H\right)=\left(\begin{array}{c} I_{C1u}\left(H\right)\\ I_{C1l}\left(H\right)\\ I_{C2\mathrm{ou}}\left(H\right)\\ I_{C2\mathrm{ol}}\left(H\right)\\ I_{C2\mathrm{iu}}\left(H\right)\\ I_{C2\mathrm{il}}\left(H\right)\\ I_{C4\mathrm{Au}}\left(H\right)\\ I_{C4\mathrm{Al}}\left(H\right)\\ I_{C4\mathrm{Bu}}\left(H\right)\\ I_{C4\mathrm{Bl}}\left(H\right)\\ I_{C4\mathrm{Cu}}\left(H\right)\\ I_{C4\mathrm{Cl}}\left(H\right)\\ I_{C4\mathrm{Du}}\left(H\right)\\ I_{C4\mathrm{Dl}}\left(H\right)\\ I_{C4\mathrm{Eu}}\left(H\right)\\ I_{C4\mathrm{El}}\left(H\right)\\ I_{C4\mathrm{Fu}}\left(H\right)\\ I_{C4\mathrm{Fl}}\left(H\right) \end{array}\right)=\left(\begin{array}{cccccccc} 1 & 0 & 0 & 0 & 0 & 0 & 0 & 0\\ 0 & 1 & 0 & 0 & 0 & 0 & 0 & 0\\ 1 & 0 & 0 & 0 & 0 & 0 & 0 & 0\\ 0 & 1 & 0 & 0 & 0 & 0 & 0 & 0\\ 1 & 0 & 0 & 0 & 0 & 0 & 0 & 0\\ 0 & 1 & 0 & 0 & 0 & 0 & 0 & 0\\ 0 & 0 & 1 & 0 & 0 & 0 & 0 & 0\\ 0 & 0 & 1 & 0 & 0 & 0 & 0 & 0\\ 0 & 0 & 0 & 1 & 0 & 0 & 0 & 0\\ 0 & 0 & 0 & 1 & 0 & 0 & 0 & 0\\ 0 & 0 & 0 & 0 & 1 & 0 & 0 & 0\\ 0 & 0 & 0 & 0 & 1 & 0 & 0 & 0\\ 0 & 0 & 0 & 0 & 0 & 1 & 0 & 0\\ 0 & 0 & 0 & 0 & 0 & 1 & 0 & 0\\ 0 & 0 & 0 & 0 & 0 & 0 & 1 & 0\\ 0 & 0 & 0 & 0 & 0 & 0 & 1 & 0\\ 0 & 0 & 0 & 0 & 0 & 0 & 0 & 1\\ 0 & 0 & 0 & 0 & 0 & 0 & 0 & 1 \end{array}\right)\left(\begin{array}{ccc} 0 & 0 & 1\\ 0 & 0 & 1\\ \cos\left(\frac{-1}{6}\pi \right) & \sin\left(\frac{-1}{6}\pi \right) & 0\\ \cos\left(\frac{1}{6}\pi \right) & \sin\left(\frac{1}{6}\pi \right) & 0\\ \cos\left(\frac{3}{6}\pi \right) & \sin\left(\frac{3}{6}\pi \right) & 0\\ \cos\left(\frac{5}{6}\pi \right) & \sin\left(\frac{5}{6}\pi \right) & 0\\ \cos\left(\frac{7}{6}\pi \right) & \sin\left(\frac{7}{6}\pi \right) & 0\\ \cos\left(\frac{9}{6}\pi \right) & \sin\left(\frac{9}{6}\pi \right) & 0 \end{array}\right)\left(\begin{array}{ccc} c_{xy} & 0 & 0\\ 0 & c_{xy} & 0\\ 0 & 0 & c_{z} \end{array}\right)\left(\begin{array}{c} H_{x}\\ H_{y}\\ H_{z} \end{array}\right)=\mathrm{MOCH}$$ (1)

The current subscripts identify the coil according to the labels given in Fig 2B, where the additional subscripts ‘u’ and ‘l’ indicate the upper and lower position in the gantry, respectively. The field is first multiplied by experimentally determined factors of the field-to-current conversion matrix **C**. Due to the symmetry of the setup, only two factors are needed, namely *c*_*xy*_ for the twelve outer coils used for the generation of *H*_*x*_ and *H*_*y*_ field components and *c*_*z*_ for the generation of the *H*_*z*_ component. Using Hall sensor measurements, these factors were determined to be *c*_*xy*_ = 1.25 A mT^-1^ μ_0_ and *c*_*z*_ = 0.25 A mT^-1^μ_0_. The current-to-orientation mapping matrix **O** then projects the desired field directions on the principal symmetry axes of the system. Finally, the orientation-to-channel mapping matrix **M** assigns these principal currents to the individual coil stacks. A linear simulation without soft-magnetic material was performed to verify that this simplified model yields the desired homogeneous fields in arbitrary orientations over the workspace. Respective field maps are displayed in Fig 2 and videos of rotating fields applied in the *xy* and *xz* plane are found in the supporting information S1 and S2 videos, respectively.

For driving of a magnetic drill, a rotating uniform field was applied about the drill axis. A uniform field **H** creates a torque **T**(**H**) on the magnetic moment **m** of the drill [18]:

$$T(H)={µ}_{0}\;m\times H={µ}_{0}\;VM\times H$$ (2)

**Fig 3:**
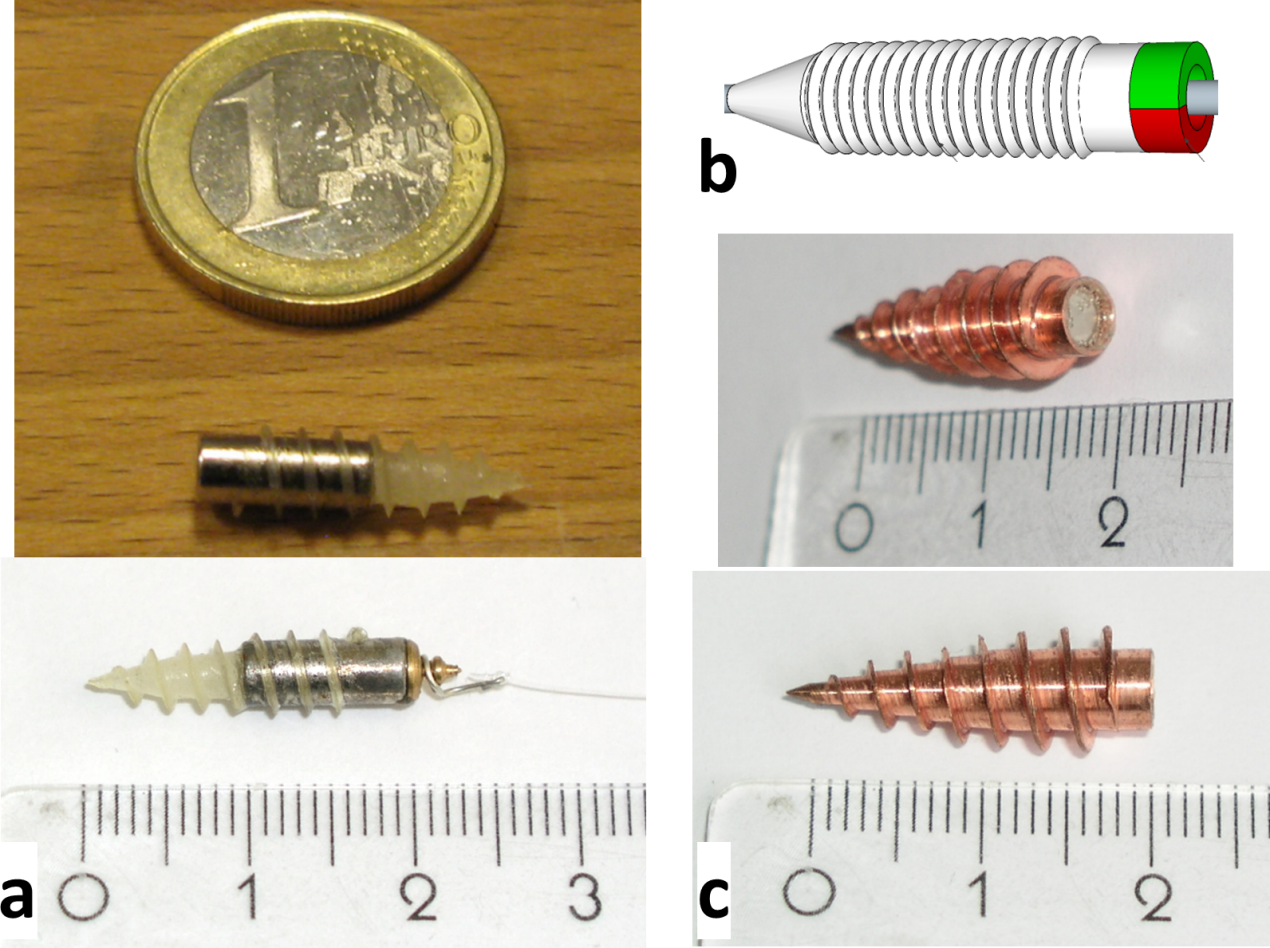
Drill demonstrators. (a) Plastic drill glued to a cylindrical NdFeB magnet, used for drilling through gel. It was fabricated by pouring epoxy resin into a self-made silicon rubber mold. For pulling a Nylon filament, a small circular plate connected to a sphere was attached to its end (bottom picture). The sphere was able to rotate freely in a wire loop attached to the filament. (b) Schematic picture of a magnetic drill or screw, showing that the magnetization is oriented transversely to the drill axis. Thus, it can be rotated by application of a field that rotates about the drill axis. (c) A copper drill with a sharp thread to be driven through tissue. The shape of the drill was generated by lathing solid copper material. Its thread was sharpened using a file. A ferromagnetic NdFeB cylinder is glued into its hollow inside.

**Fig 4:**
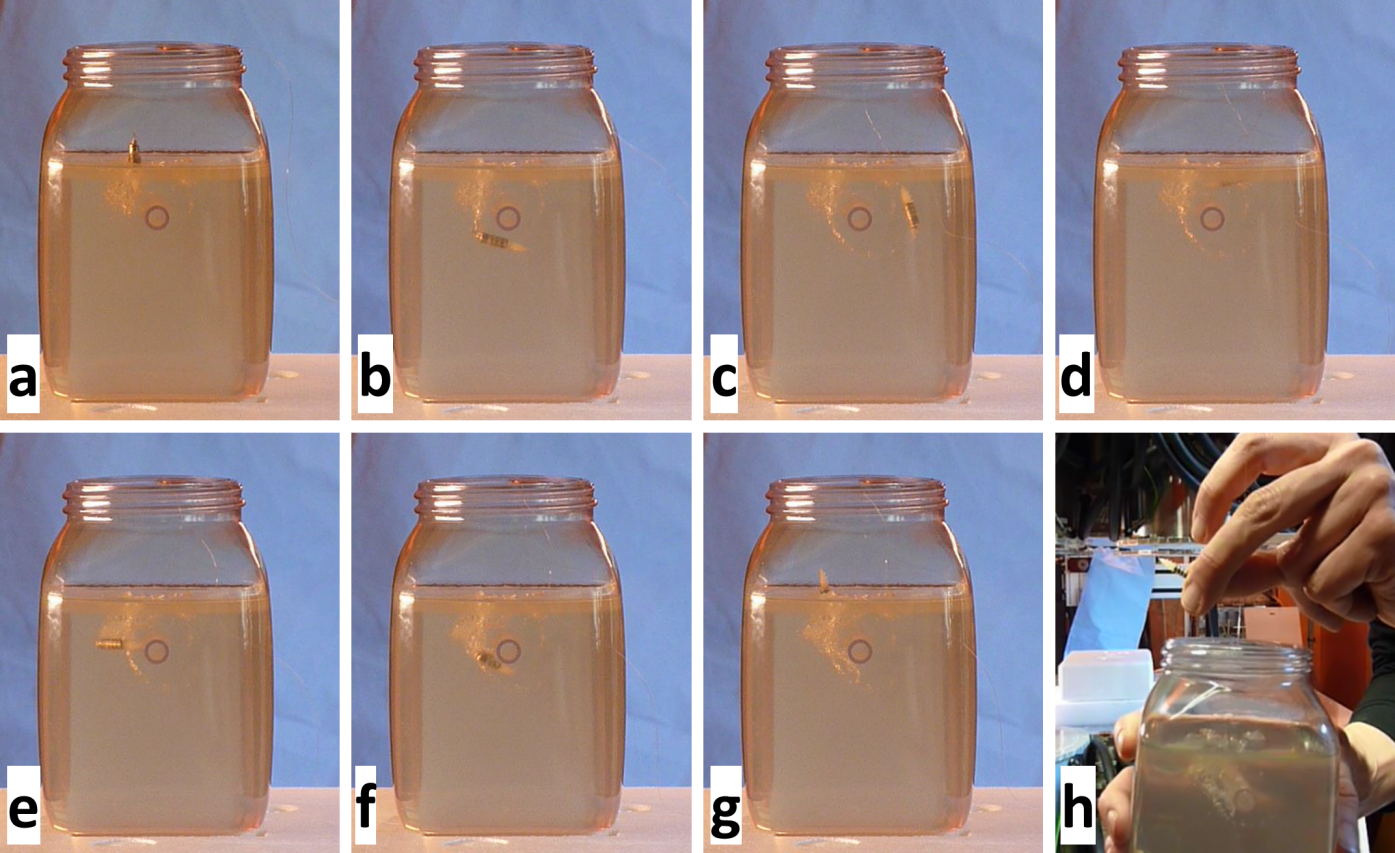
Tying a knot in gel by pulling a filament with a magnetic drill. The magnetic field direction rotates about the drill axis for driving the drill (cf. Fig 3A) in the gel. To force the drill on a curved trajectory, the axis of field rotation is changed in small steps so that the drill axis can follow. At first, a loop is laid out (a-d), then the drill follows a tight curve around the initial vertical segment of the path (e) so that it can enter (f) and pass through the loop to complete the knot (g). The circular structure at the center is the front view on a rod, around which the knot closes (h). The full video can be found in S3 video.

**Fig 5:**
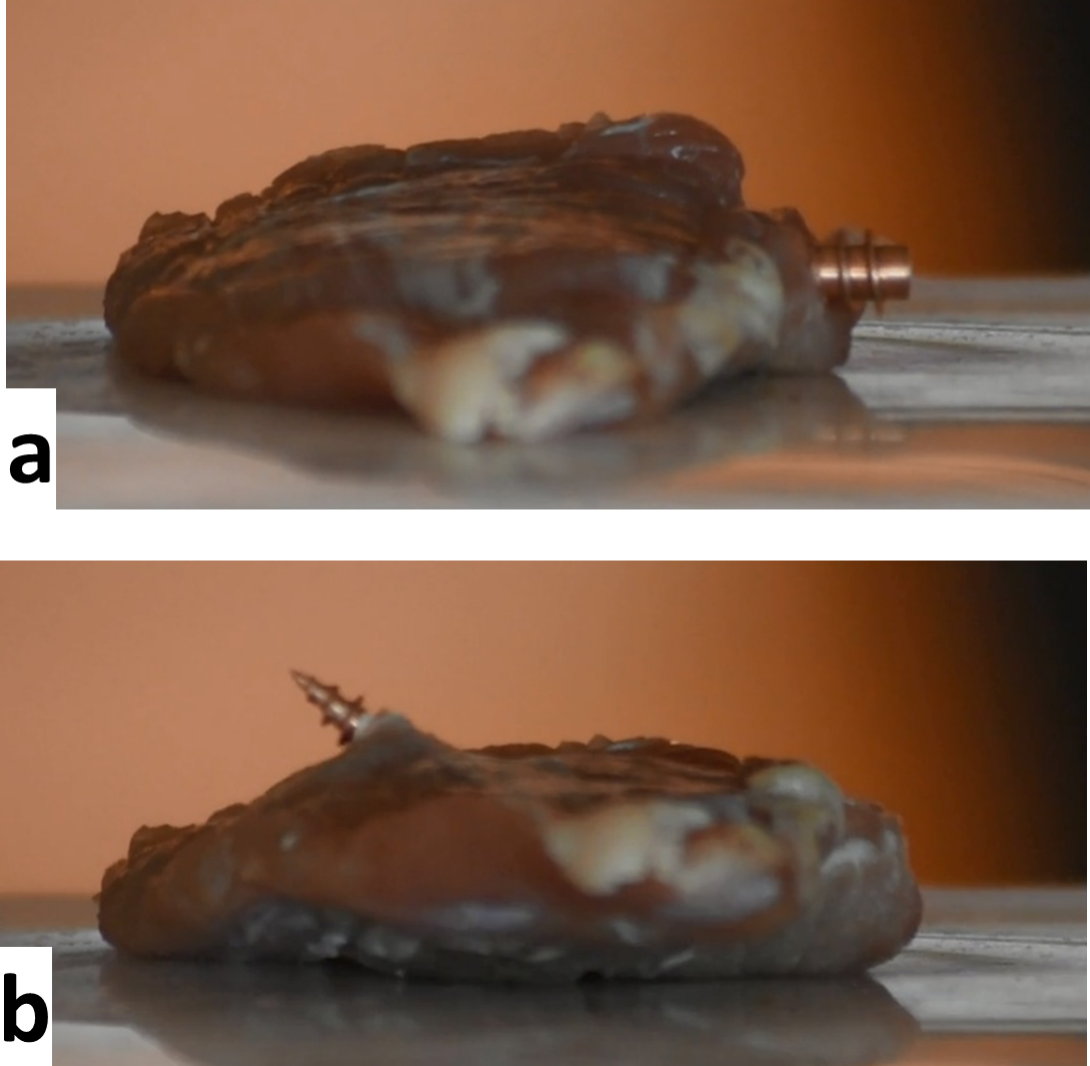
Driving a drill through a raw pork fillet. The trajectory starts horizontally (a) and is slightly curved upwards, so that the drill exits towards the top (b). The full S4 video in the supporting information shows that the drill encounters increased resistance when passing through tendon fibers.

The magnetic moment is given by the product between volume *V* and magnetization **M** of the magnetic material. Permanent magnets are employed for the drills and the orientation of the magnetic moment is chosen to be perpendicular to the drill axis (cf. Fig 3B), so that the torque vector aligns with the drill axis and induces a rotation [31]. The required currents for generating the rotating field sequence were calculated according to Eq 1. One rotation cycle was typically discretized into 32 to 128 current steps with duration 10.2 ms per step. For steering a drill on a curved trajectory (cf. Fig 4 and Fig 5), the plane of field rotation was slightly tilted after each rotation cycle. The drill axis always aligns perpendicularly to the plane and thus follows the changing orientation of the plane as long as the tilting step is not too large.

The maximum homogeneous field strengths that the field generator achieves are about 100 mTμ_0_^−1^ in the *xy* plane and up to 400 mTμ_0_^−1^ in *z* direction. In principle, field amplitudes can be varied with frequencies up to 100 Hz, however, for high amplitude changes, limited slew rates reduce the achievable frequencies to less than 10 Hz. The homogeneous field strengths applied in the reported experiments ranged from 30 to 90 mTμ_0_^−1^ at frequencies up to 10 Hz.

For spatially selective magnetic actuation [31] and for MPI [34], a strong gradient field or “selection field” was generated by feeding the upper and lower innermost (C1) and ring-shaped coils (C2) with opposing currents. This corresponds to inverting the sign in the second row of the current-to-orientation mapping matrix **O** in the field-to-current conversion. At the above-mentioned maximum currents, a gradient of more than 3.0 Tm^-1^μ_0_^−1^ can be achieved in vertical direction. Field gradients can be used to generate forces on magnetic objects. The force **F** depends on the magnetic moment of the device **m** and on the gradient of the field **H** [18]:

$$F={µ}_{0}(m∙\nabla )H{=µ}_{0}V(M∙\nabla )H$$ (3)

In MPI, a high selection field gradient ensures high spatial resolution [35]. For continuous operation, the currents are reduced to about 60 A, leading to a vertical field gradient of 2.0 Tm^-1^μ_0_^−1^. At this gradient strength, the resulting field-free point (FFP) can be moved along a vertical line of length 20 cm by applying asymmetric currents to the center coils. By adding homogeneous lateral fields, the FFP can be moved within a sphere of diameter 20 cm without reduction in gradient strength (see Fig 1A). Homogeneous fields are also called “focus fields” as they shift the focus of the imaging volume in space.

Field and field gradient values were determined by measuring local field amplitudes with a Hall sensor array consisting of four 3D sensors positioned at selected corners of a cube of edge length 18 mm. To verify that the desired values were achieved over the complete workspace, the sensor array was moved to a set of well-defined 3D positions using a robot. For the reported initial imaging experiments, reduced gradient strengths of only up to *G*_*z*_ = 0.8 Tm^-1^μ_0_^−1^ were applied to achieve a useful spatial coverage despite the limited drive field amplitudes reported below.

Two of the reported experiments on the more sophisticated magnetic devices were performed on a pre-clinical field applicator, which can generate similar field configurations as the described clinical-scale system, however with a simpler field applicator and a smaller bore with a diameter of 12 cm [36].

### Magnetic objects

Several helical objects were built, all with magnetization transverse to the axis of rotation. Fig 3 displays magnetic drills made for untethered operation in gel or tissue.

In Fig 3A, a drill made from a FeNdB permanent magnet and epoxy resin is displayed. For manufacturing, a mold of a screw was made using silicone rubber. After hardening and removal of the screw, the mold was filled with epoxy resin and a magnet was placed in the hole, too. After extracting the hardened drill, a brass plate was glued to the flat end. At the center of the plate, a cylinder with a spherical end piece was machined. Around the cylinder, a piece of silver plated copper wire was wound and attached to a monofilament polyamide thread. The purpose of the mechanism is to form a bearing around which the piece of wire can rotate so that the thread is not twisted while being pulled, allowing smooth operation of the drill. The diameter of the magnet was 4 mm and the length was 10 mm. Fig 3B shows the transverse orientation of the magnetization with respect to the long axis of the drill. In Fig 3C, a machined drill for cutting through tougher material is shown. It is made from pure copper and uses the same kind of permanent magnet as the drill in Fig 3A, which is glued into the cylindrical cavity inside the drill. The drill was machined in a way that the thread would become as sharp as possible and it was further sharpened using a file. No quantitative measure of the sharpness was performed. However, it seemed not to be particularly sharp: the screw did not cut into the finger when pinched with maximum force and then rolled between the fingers.

**Fig 6:**
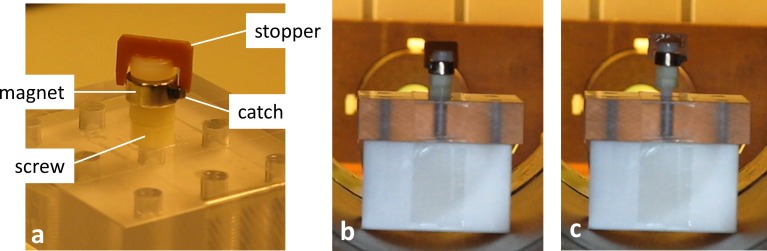
Impulse drive demonstration. (a) Impulse drive mechanism demonstrator attached to a screw. The magnetic ring (with magnetization perpendicular to the screw axis) can rotate freely about the axis. It follows an applied rotating field until the black catch is blocked by the stopper. After the magnetic field rotation has further advanced by more than 180 degrees, the ring magnetization realigns with the field and the ring snaps back until the stopper blocks the catch on the other side. Due to the fast ring rotation while snapping back, a large angular momentum is transferred to the stopper, which is attached to the screw and thus gives an impulse to loosen or tighten the screw. The applied rotational field is able to loosen a tightly fixed screw in the impulse or high-torque mode (b). Once loosened, the screw can be driven upwards using in inverted sense of field rotation (c). The full video can be found in S5 video.

In Fig 6, a screw with an impulse drive mechanism is shown. The impulse drive consists of a ring magnet (magnetized transversely to the rotational axis) mounted on a screw (metric, M5, polyamide). The permanent magnet can rotate on the screw axis, i.e. the two nuts below the magnet are tightened together and they do not clamp the magnet. To the magnet, a simple iron cube is attached by gluing and magnetic forces. This cube together with a fiber-enforced plate forms a stopping mechanism. Thereby, the rotation of the magnet is limited to slightly less than 180°. The simplest way to operate the screw is to apply a rotating magnetic field, with the axis of rotation aligned with the screw axis. The field rotates the ring magnet until it reaches the stop position. For the next 180° of rotation, the magnet remains stopped if the applied torque is too low to rotate the whole screw. After 180°, when the torque on the magnet reverses sign, the ring snaps back and hits the second stop position. This hit produces a high torque on a rigidly fixed screw. The mechanism can be applied to tighten or loosen a rigidly fixed screw. A loose screw can be rotated continuously with a lower torque by pushing the stop by a rotating magnetic field. Note that the sense of rotation in impulse mode is opposite to the one in low torque mode.

**Fig 7:**
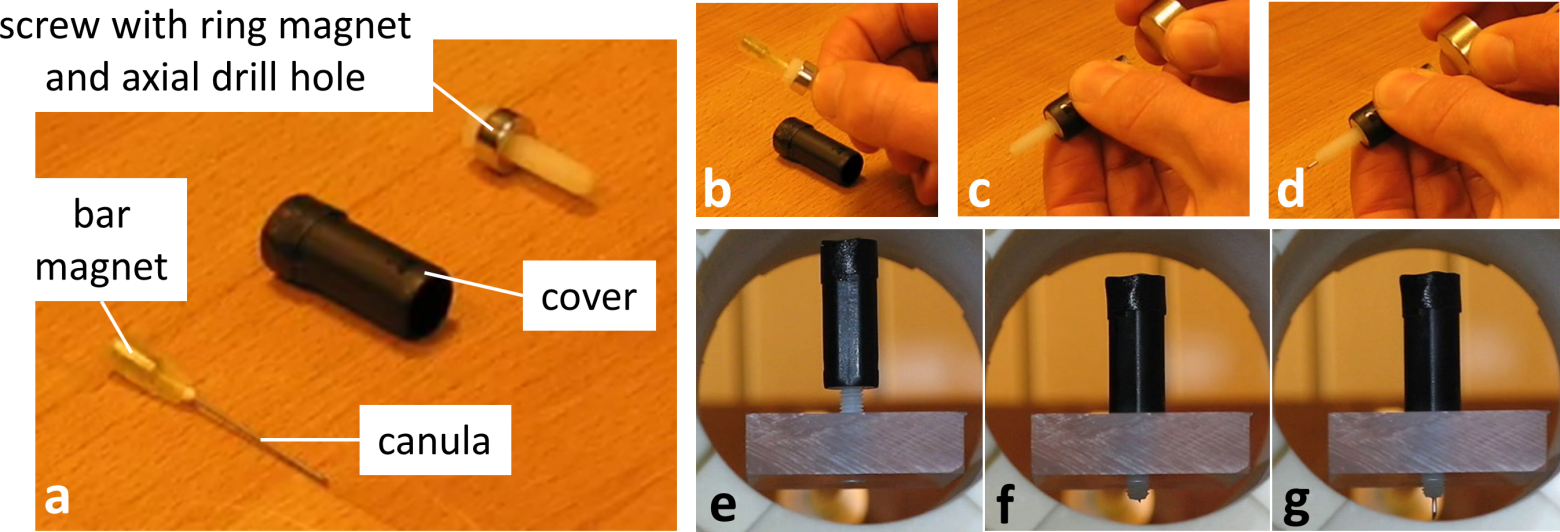
Magnetic screw with magnetically actuated needle as a demonstrator for a potential biopsy taking or particle/drug delivery device. (a) The needle is attached to a bar magnet and can be inserted into the central drill hole in the screw (b). A cover is attached to the ring magnet that is fixed to the screw. Application of a field gradient with field orientation parallel (c) or anti-parallel (d) to the bar magnet pushed the needle in or out of the screw, respectively. (e) A rotating horizontal field drives the screw into the screw hole (f). Application of a field gradient pushes the needle out of the screw (g). Inverting the field enables pulling back the needle (not shown). A video of the procedure is provided in the supporting information S6 video.

In Fig 7A, a model of a more complex device is shown. It can be steered to a target position by screwing or drilling. There, a needle can be pushed out of the screw, e.g. to be inserted into tissue. The device consisted of a screw (metric, M5, polyester) with a rigidly attached ring magnet with permanent magnetization transverse to the screw axis. Along the screw axis, a hole was drilled for insertion of an injection needle. In the connector of the needle, a further permanent magnet was placed and fixed with adhesive. Its magnetization was oriented parallel to the screw/needle axis, i.e. orthogonal to the magnetization of the ring magnet. Over the screw head and the needle magnet, a cover made from adhesive tape was placed to keep the needle from falling out while still providing the ability to move. Using homogeneous rotating fields, a torque can be created on the ring magnet to advance the screw, while using a gradient field, a force can be generated on the needle to push its tip out of the device (cf. Fig 7C and 7D). For demonstration, the device was screwed into a thread in a plastic plate and then operated by magnetic fields.

The individual appearing in the supporting information of this manuscript has given written informed consent (as outlined in PLOS consent form) to publish the video material.

### Imaging system and imaging experiments

For imaging, the scanner is equipped with a three-channel drive coil, as depicted in Fig 1A and 1C. For the *x* direction, a solenoid coil is used, whereas for the *y* and *z* direction, saddle coils were employed. Signal excitation in 3D real-time MPI is achieved by driving the FFP on a 3D Lissajous trajectory through the application of orthogonal homogeneous drive fields at three different frequencies [37]. By use of matching circuits, the resonance frequencies of the three drive field channels were tuned to *f*_*x*_ = 150.4 kHz, *f*_*y*_ = 157.2 kHz, and *f*_*z*_ = 144.1 kHz, respectively. These frequencies were derived from a common frequency by division by 23, 22, and 24, respectively. The resulting repetition time of the closed 3D Lissajous trajectory amounted to *TR* = 1.76 ms. The three channels were driven by custom-built class D amplifiers, whose output was passed through several filter stages to suppress harmonics of the excitation frequencies. The oil-cooled drive coils were designed to deliver a maximum field amplitude of 10 mTμ_0_^−1^ on the *x* axis and 8 mTμ_0_^−1^ on the *y* and *z* axis.

The drive coils were also used for signal reception. After removal of the excitation bands using notch filters and subsequent high-pass filtering at about 200 kHz, the signal of each channel was amplified by a low-noise amplifier and digitized using a 16 bit ADC. An image was reconstructed from the data acquired during one *TR* by iteratively solving the imaging equation [38] using a system function matrix acquired beforehand in a calibration scan [39]. In the experiments reported here, only one (*x*) or two (*x* and *z*) drive channels were employed simultaneously.

For an initial 2D imaging experiment on a ‘P’ shaped phantom, a gradient of *G*_*x*_ = *G*_*y*_ = 0.2 Tm^-1^μ_0_^−1^ and *G*_*z*_ = 0.4 Tm^-1^μ_0_^−1^ was applied and the FFP was moved along the *x* direction by application of a drive field with amplitude 6 mTμ_0_^−1^, leading to a spatial coverage of 60 mm. In *z* direction, the FFP was moved by applying asymmetric currents to the C1 and C2 coils, i.e., by superimposing focus fields on the selection field. Thus, the encoded *x* line was shifted in *z* direction in 19 steps with a spacing of 5 mm over the distance of 95 mm. Using this method, a 2D image of the phantom was acquired in roughly 8 seconds, with an averaging time per line of 400 ms. The ‘P’-shaped tube phantom had an inner diameter of 1.6 mm that was filled with Resovist at a dilution of 1:12 (~ 40 mmol(Fe)l^-1^).

For an initial 3D imaging experiment of a spiral-shaped phantom, 1D drive field encoding along *x* was combined with a sinusoidal focus field shift along *y* and robot motion of the phantom along *z*. Both the drive field and the focus field amplitude were 8 mTμ_0_^−1^ and the focus field frequency was 20 Hz. The gradient strength was the same as in the previous experiment, so that an *xy* coverage of 80 mm × 80 mm resulted with a 2D imaging time of 50 ms. In *z* direction, the imaging plane was shifted over 19 positions with a spacing of 4 mm. The phantom was a flexible tube with an inner diameter of 1.6 mm filled with Resovist at a dilution of 1:10 (50 mmol(Fe)l^-1^). The tube was wound in a spiral around a sphere with a diameter of 50 mm. Reconstruction was performed slice by slice using a system function acquired beforehand on a 2D grid of 31 × 31 with a spacing of 3.2 mm × 3.2 mm.

For a 2D real-time imaging experiment, amplitudes of *H*_*x*_ = *H*_*z*_ = 4.0 mTμ_0_^−1^ and gradient strengths of *G*_*x*_ = 0.4 Tm^-1^μ_0_^−1^ and *G*_*z*_ = 0.8 Tm^-1^μ_0_^−1^ encoded a rectangular region of 20 mm × 10 mm in the *xz* plane. 28 Lissajous cycles were averaged for improving SNR, resulting in a temporal resolution of 49.28 ms. For system calibration, a 3D grid of dimensions 13 × 3 × 11 centered on the plane of FFP motion was covered by moving the sample in steps of 4.0 × 4.0 × 2.0 mm^3^ using a robot. Imaging and system calibration were performed with a point sample of 100 μl of pure Resovist at 500 mmol(Fe)l^-1^ (Bayer Healthcare, Germany).

## Results

Steering of an untethered magnetic drill was demonstrated in a gel phantom (Fig 4) and a tissue sample (fillet of pork, Fig 5), respectively.

For a demonstration of complex 3D steering, a drill (cf. Fig 3A) pulled a filament through a gel phantom and formed a knot around a rod positioned in the gel. The minimal curvature radius was about 2 cm and thus roughly corresponds to the length of the screw. Images acquired at different time points of the sequence are displayed in Fig 4. The full sequence can be found in S3 video. The field sequence was precomputed based on the desired trajectory and the experimentally determined propulsion speed of the drill in gel. The field amplitude was 30 mTμ_0_^−1^. The drill rotation frequency was 3 Hz. After each full rotation, the orientation of the plane of field rotation was changed in small steps (≤ 7 degrees) to force the drill on a curved trajectory.

For a demonstration of the high torque magnitude that can be achieved, the copper drill (cf. Fig 3C) was driven through a fillet of pork. A simple trajectory was chosen that started in horizontal direction and then was curved upwards. Before the start of the field sequence, the drill was drilled manually into the tissue, deep enough to be safely held in place by the tissue (cf. Fig 5A). For rotation, a field amplitude of about 90 mTμ_0_^−1^ was applied with a rotation frequency of 0.77 Hz. The drill cuts through the tissue and eventually leaves the fillet towards the top on the other side (cf. Fig 5B). The tissue sample consisted mainly of muscle, but some connective tissue was present as well. Most of the time, the drill was able to directly penetrate the connective tissue, but sometimes strands of connective tissue wound around the screw and temporarily blocked the rotation, leading to a motion of the whole fillet (see S4 video).

One way to increase the torque and hence tissue penetration capability is to use an impulse drive mechanism. The experimental impulse drill described above was tested in the pre-clinical field applicator, as shown in Fig 6. For this demonstration, the mechanism was screwed into a plastic plate employing maximum manual force. This assembly was placed in the scanner and a rotational sequence was applied in the transverse plane with field amplitude 60 mTμ_0_^−1^ and rotation frequency 1.5 Hz. The pulsed operation always managed to loosen the blocked screw. After untightening of the screw, the sense of field rotation was reversed to unscrew the device out of the thread.

In Fig 7E–7G and S6 video, remote operation of the needle-pushing device is demonstrated in the pre-clinical field applicator. First, a rotating field of 15 mTμ_0_^−1^ with a rotation frequency of 0.6 Hz was employed to screw the assembly into the plate. After a few rotations, this motion was stopped, and a gradient field of 1.0 Tm^-1^μ_0_^−1^ was employed, resulting in a rapid forward movement of the needle and exposing a few mm of the needle tip as displayed in Fig 7G.

**Fig 8:**
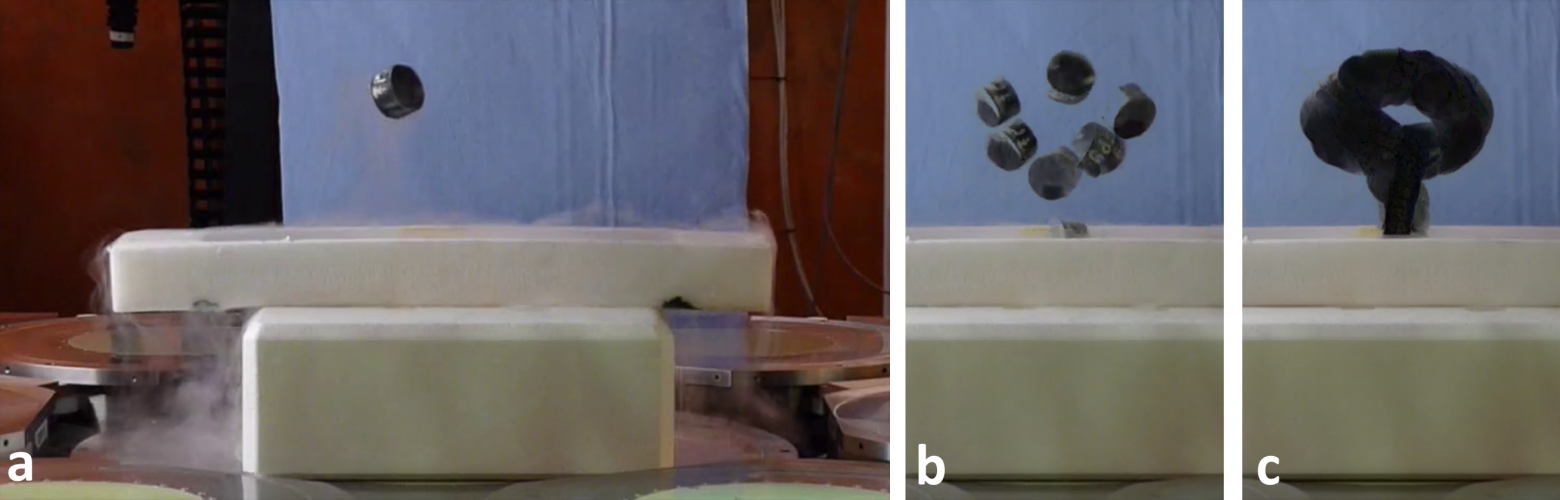
Demonstration of steering the field-free point (FFP). A superconductor is a perfect diamagnet and minimizes its energy by moving to the FFP. The energy gain is large enough to provide the required potential energy for levitating the superconductor (a). While following the FFP, the superconductor rotates freely, as seen in the overlay of several frames extracted from a video sequence (b). A minimal intensity overlay of all video frames delineates the FFP path consisting of a linear segment for lifting the superconductor out of the liquid nitrogen bath and an elliptical path (c). The video can be found in supporting information S7 video.

In Fig 8, the ability to control the large scale movement of the FFP is visualized by levitating a superconductor. An YBCO type superconductor was placed in a vessel inside the large field applicator. The superconductor was cooled to reach superconductivity by filling the vessel with liquid nitrogen. Then the gradient was slowly ramped up to *G*_*z*_ = 2.2 Tm^-1^μ_0_^−1^ with asymmetric currents on the *z* coils to bring the FFP close to the superconductor’s rest position. Using focus fields, the FFP was then moved on a pre-defined trajectory, lifting the magnet, moving it twice on an elliptical path, and eventually placing it back at the original position. The superconductor hovered a few centimeters below the field free point, slowly increasing the distance due to progressive heating and partial break down of the superconductivity.

**Fig 9:**
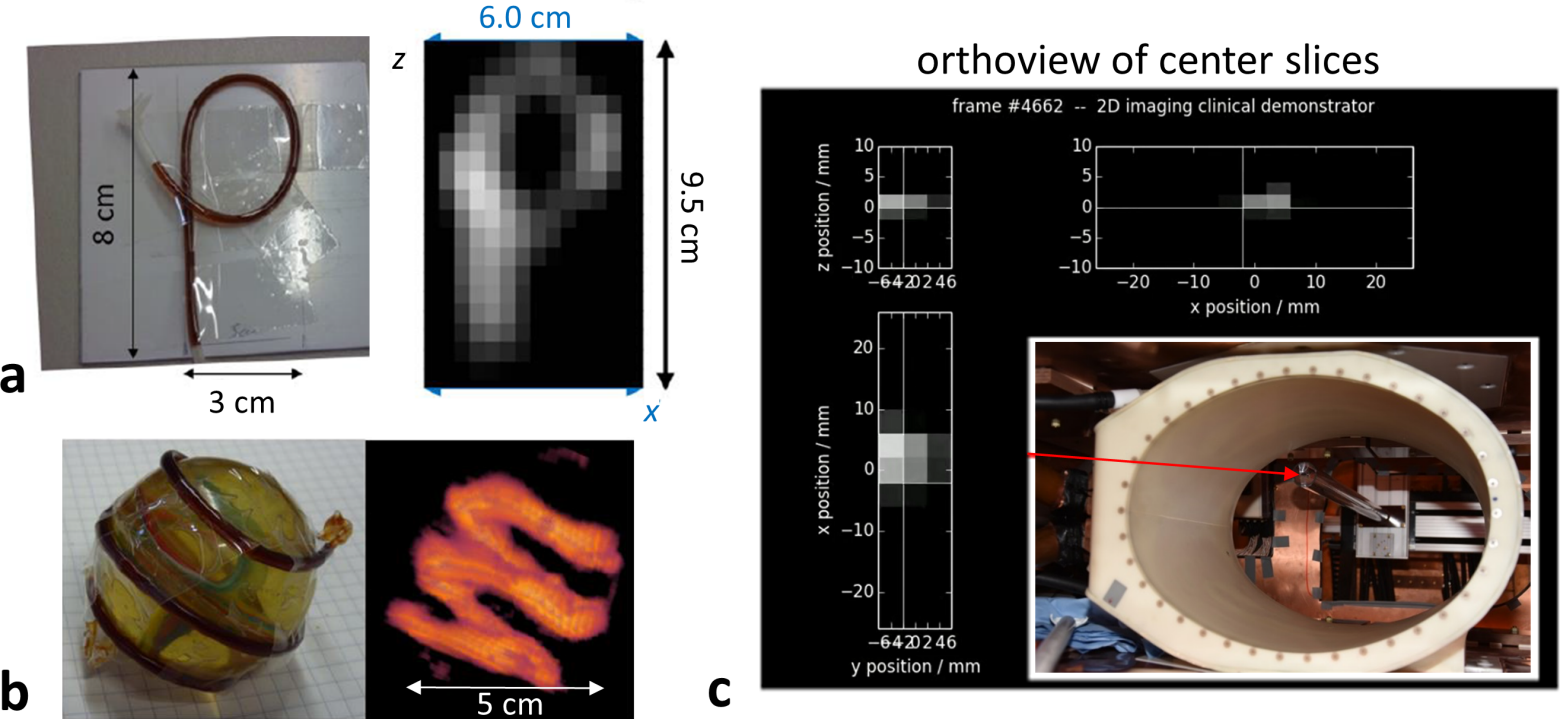
Initial imaging experiments on a clinical-scale system. (a) 2D imaging by a combination of 1D drive field excitation and 1D robot-based translation of the phantom. (b) 3D imaging by combining 1D drive field excitation with 1D fast-focus field excitation and 1D robot motion. (c) 2D real-time imaging by 2D drive field excitation.

In Fig 9, images from three initial imaging experiments on the clinical scale system are shown. Fig 9A displays the 2D image acquired from the ‘P’ shaped phantom. The reconstructed voxel size was 4 mm and 5 mm in *x* (horizontal) and *z* (vertical) direction, respectively. The half-width of the measured signal profile of the Resovist-filled tube was about 8 mm. As the tube diameter was much smaller, this value corresponds to the achieved spatial resolution. Fig 9B displays a surface-rendered view of the 3D image acquired of the spiral phantom using both robot motion in *z* direction and fast focus field variation along *y*. At an acquisition time per *xy* slice of 50 ms, the observed resolution was about 6 mm in the slice. Fig 9C displays orthogonal slices through the 3D data set reconstructed from the point sample excited by a 2D drive field sequence. Here, twice the gradient strength is employed when compared to the two previous experiments, leading to an improved spatial resolution, which is estimated to be about 4 mm in *x* and about 2 mm in *z* direction. The sample motion can be observed in the corresponding S8 video.

## Discussion

The experiments demonstrate that 3D remote control of untethered drills is feasible over a clinical scale workspace (Figs 3–5). The torques achieved using rotating uniform fields are strong enough to drive a drill through tissue. In order to employ drilling devices in minimally invasive surgery, smaller objects may be necessary. As the magnetic moment and thus the torque scales with the volume of the magnetic material (cf. Eq 2), smaller devices are limited in the achievable torque. Fortunately, friction-induced torques also scale with the volume, as they are determined by the surface area of the device multiplied by the length of the lever arm. Therefore, operation of magnetic machines using torques works well at all size scales and can be used even for machines of a few microns in size [40]. Although torques were large enough for cutting through tissue, higher torques may be desired in some applications. Here, a miniaturized version of the demonstrated impulse drive mechanism shown in Fig 6 can be employed to strongly increase the maximum torque. We estimate that, depending on the implementation of the impulse drive mechanism, the torque can be increased by more than an order of magnitude in fairly rigid material. Thus, also smaller drills could pass through tissue even in the presence of tendons. Torques generated using the impulse drill mechanism may even be sufficient to drive the untethered drill into bones.

In S3 and S4 videos, one can observe that the drills wobble slightly about their axis of rotation when moving on a curved path and their axis orientation may lag behind the changing axis of rotation of the applied field. Several mechanisms contribute to this. First, the orientation of the rotating field axis was updated in discrete steps of up to 7 degrees after each rotation. With smaller steps, smoother steering can be expected. To this end, the orientation could be updated already during a single rotation or drills with a smaller pitch could be used in order to have less propulsion per rotation and thus more control. Second, the simple linear field-to-current model leads to the generation of fields that deviate from the desired fields. This is especially problematic when the plane of field rotation leaves the horizontal plane. The reduced symmetry then may lead to a field amplitude variation during rotation and thus a variable rotation speed. Also, due to the closer distance to the field generators in *z* direction, larger field variations with position occur, which can already be seen in the linear field simulation shown in S2 video. Third, asymmetric friction forces act on the drill when driven on a curved path. Due to the higher friction at one side, the drill axis can leave the plane containing the curved trajectory segment, leading to deviations from the desired path. In our experiments, these deviations were corrected based on visual control. For in-body application, this feedback information must be delivered by a fast medical imaging approach as discussed below. With image guidance, untethered drills could be used to carry drugs for local drug delivery [16] or to pull wires for better placement of RF leads in tumor ablation treatments.

The high flexibility of the field applicators enables switching between uniform and gradient fields or arbitrary field superpositions. This can be used to actuate different mechanisms of devices that respond to either torque or force. The needle-pushing device (Fig 7) could be developed into an untethered drill for minimally invasive biopsy acquisition at locations that are hard to reach using a needle from the outside. A drill could be driven through tissue to a site of interest by uniform rotating fields. At its destination, gradient fields could be employed to push the biopsy needle into the tissue and to retract it into the device. Then the drill could be driven back to its entrance point by applying the initial rotating fields with inverted sense of rotation.

The superposition of field gradient and homogeneous fields furthermore enables spatially selective remote actuation of helical devices [31]. This is a new approach to selectively address individual members of a team of micro-machines based on their spatial position. In a local drug delivery scenario, one could envisage a swarm of micro-machines, of which only those release their drug load, which have reached a desired position. Also, small micro-machines could be devised that are actuated to shield or de-shield an internal radiation source and thus can deliver remotely controlled adaptive internal radiation therapy.

Using gradient fields, substantial forces can be created, as demonstrated in the levitation experiment of the superconductor (Fig 8). The force is proportional to the magnetization (cf. Eq 3), which in the case of a superconductor corresponds to the field strength of the local field that is expelled from the superconducting material. For the superconductor (and all diamagnetic materials), the magnetization direction is opposite to the field direction and the force points into the direction of decreasing field, i.e., towards the FFP. In the levitation experiment, the superconductor does not reach the FFP, but remains at a position where the field is strong enough to create a force that compensates the gravitational force. For real applications, ferromagnetic materials need to be used, which experience a force that points away from the FFP. In contrast to diamagnetic objects, a ferromagnetic object therefore does not reach a stable equilibrium position. The object either needs to be attached, e.g. to a catheter in catheter steering applications [6], or, if untethered, requires active closed-loop steering of the applied fields based on feedback using a fast imaging method [14,25]. For both applications, the imaging capabilities of the MPI system itself can be employed [32,33]. In the cited MPI-based steering applications, soft magnetic devices have been used. The magnetization of these objects depends on their distance to the FFP and thus the force can be controlled very finely via positioning of the FFP, while a high gradient strength can be maintained for concurrent MPI localization [32]. When scaling down force operated devices, forces drop with the volume, while viscous drag only drops with the linear dimension (laminar flow). For compensation, force operation of miniature devices requires higher magnetization. As saturation of the soft magnetic material is not achieved within the imaging workspace, the full magnetization potential of the objects is not exploited. Using additional homogeneous fields, the material could be driven into saturation. However, this would require replacing concurrent MPI localization with interleaved localization and force application intervals. For reaching saturation magnetization, the high homogeneous fields available in MRI machines may be an alternative [13], however, generation of high force-inducing gradients leads to field distortions that compromise concurrent force application and MR imaging [41]. To be able to make use of MRI’s superior tissue contrast for localization of magnetic material in the body, interleaved imaging and magnetic manipulation approaches have been suggested that either use mechanical repositioning of gradient-amplifying magnetic material [42] or of the patient within the fringe field of the MRI main field [43]. To mitigate low magnetization of soft-magnetic material in our field configuration, hard magnetic devices would be an alternative option, however, common hard magnetic materials have lower saturation magnetizations (~ 1.45 T for NdFeB) than soft magnetic materials (~ 2.43 T for FeCo). In view of these limitations, torque-based propulsion of helical devices using rotating fields seems to be a more powerful alternative for navigation of micro-scale machines than application of forces [2].

While to present, closed-loop MPI based steering has only been demonstrated on a small pre-clinical system, the imaging experiments reported here indicate that there is no principle limitation to image guided steering on a clinical-scale system. The limited spatial resolution of MPI of currently 1.5 to 3.0 mm for imaging of nanoparticles should not be a limiting factor for tracking of individual point-like objects: a constrained reconstruction assuming a single point-like object in combination with the steeper magnetization curve of a magnetic marker [32] has the potential of delivering submillimeter resolution. The necessary separation of marker signal from concurrently measured nano-particle signal has been demonstrated using multi-color MPI [29].

Scaling an MPI system from pre-clinical to clinical size creates a number of challenges. One is the requirement to maintain a high gradient strength. In MPI, gradient strength *G* is inversely proportional to spatial resolution *Δx*, and to reach a spatial resolution of a few millimeters with existing tracer materials, a gradient strength of more than 1 Tm^-1^μ_0_^−1^ is required [35]. The levitation experiment of the superconductor shows that a gradient strength above 2 Tm^-1^μ_0_^−1^ can be achieved in vertical direction while the FFP is moved over a workspace with a diameter of about 20 cm. For real-time imaging, a second challenge is the generation of sufficiently large drive fields in all three spatial directions. The drive fields operate in the kHz range and thus enable very fast 3D spatial coverage of typically tens of volumes per second [37]. The spatial coverage is determined by the range over which the FFP can be shifted by the applied drive field amplitudes in the presence of the selection field gradient, e.g. over Δ*x* = 2*H*_*x*_ *G*_*x*_^-1^, for a drive field amplitude of *H*_*x*_ applied on the *x* channel. While small scale systems can generate drive field amplitudes of about 20 mTμ_0_^−1^, power dissipation limits amplitudes to about 10 mTμ_0_^−1^ in a clinical scale system. In clinical applications, the most relevant limitation to the applicable drive field amplitudes is the peripheral nerve stimulation created by the time-varying fields in a patient [44]. A volunteer study indicated that stimulation thresholds are around 5 mTμ_0_^−1^, with a slight dependence on the spatial direction of the field [45,46], but can be increased when increasing the frequency from 25 to 150 kHz. Our clinical system operates at frequencies around 150 kHz with amplitudes around 6 mTμ_0_^−1^, in order to maximize the voxel acquisition rate, while staying below approved safety limits for both nerve stimulation and patient heating [46]. With 6 mTμ_0_^−1^ on all axes and a gradient strength of 2 Tm^-1^μ_0_^−1^ in *z* direction, the spatial coverage with the drive fields alone is small, only 12 mm × 12 mm × 6 mm. However, this volume is scanned with a high repetition rate of 568 3D-images per second, since the image acquisition time is only 1.76 ms. The focus fields can be used to shift this volume in space, but the shift speed is also limited. Therefore, it would be challenging to achieve full high-resolution 3D heart coverage with more than a few volumes per second. Here, improved tracer materials with a steeper magnetization curve need to be developed [47], so that the resolution requirement can be satisfied with a lower gradient strength. Alternatively, the resolution loss is tolerated and low gradient imaging is only used for overview imaging to identify the smaller volumes of interest to be imaged at full temporal and spatial resolution. For tracking magnetic point-like devices, the combination of low gradient overview and high gradient zoom imaging would be sufficient. The available volume imaging rates would also enable fast closed-loop control of the applied fields, e.g., to adapt to a changing device position due to motion or blood flow. For device visualization, either the negative contrast of the artifact created by the magnetic material or the positive contrast of dedicated nano-particle markers can be employed [33].

The results reported here indicate that clinical scale remote steering of devices as well as fast imaging are in fact feasible. On the imaging side, further investigations are necessary to assess to what degree high imaging speed, large spatial coverage, and good resolution can be achieved in a single measurement, or whether multiple measurements with different trade-offs need to be employed. Moreover, although we estimate that imaging would be feasible at concentrations of iron oxide materials that are clinically approved for MRI [37,48], the sensitivity of the system was not yet optimized and its ultimate sensitivity limits are not yet known.

How do the envisioned capabilities of an MPI-based magnetic steering system compare with existing approaches? Dedicated clinical-scale electromagnetic actuation systems rely on X-ray imaging for tracking [23] and thus expose the patient to ionizing radiation, which is not optimal for longer steering procedures. Both MRI and MPI have the advantage of providing radiation-free device localization information for closed-loop steering. While MPI is an emerging imaging modality, MRI is clinically established and has the benefit of high availability. It furthermore provides excellent native soft-tissue contrast at high spatial resolution without the need for a tracer material. Technically, MRI systems can apply forces using gradient fields and the force magnitude benefits from the high magnetization of magnetic material induced by the high field strength of the MRI main field (typically 1.5 T or 3.0 T). On the other hand, the fixed orientation of the main field prohibits controlled application of torques. Force-based manipulation is well suited for manipulation of millimeter-sized objects in the blood stream using closed loop steering [26]. Approaches for increasing gradient strength like magnetic dipole navigation [41] or fringe field navigation [43] enable a size reduction of the magnetic objects and thus increase the potential of MRI for catheter navigation or targeted delivery of drugs using magnetic carriers. Regarding force-based actuation, the MPI system provides higher switchable gradients than MRI machines but possibly lower magnetization levels and thus may not bring a substantial improvement, except for a faster 3D device localization. What the MPI field applicator offers, however, is torque-based manipulation. With individual millimeter-scale objects, we demonstrated 3D controlled propulsion in dense tissue, which may be useful for minimally invasive surgery. At sub-millimeter scales, torque-based propulsion is inherently more efficient than force-based propulsion [2], so that the system could be applied to the manipulation of micro-machines. In addition, the MPI field generator adds the possibility of combining gradient fields with homogeneous fields. On the one hand, this enables control of mixed force and torque operated devices (as demonstrated using the needle mechanism), on the other hand, it adds new functionality like the spatially selective actuation of helical micro-machines [31].

## Conclusions

Our magnetic field applicator demonstrates that a very high degree of control over magnetic devices can be achieved at the clinical scale. Together with its potential for magnetic particle imaging, this technology represents a powerful platform for the exploration of new minimally invasive procedures under real-time imaging control.

## Supporting information

S1 video Animation of simulated rotating uniform field. The field vectors rotate in the horizontal or *xy* plane (cf. Fig 2C).
(MP4)

S2 video Animation of simulated rotating uniform field. The field vectors rotate in the vertical *xz* plane (cf. Fig 2D).
(MP4)

S3 video Tying a knot in gel by pulling a filament with a magnetic drill (cf. Fig 4). (MP4)

S4 video Driving a drill through a raw pork fillet (cf. Fig 5). (MP4)

S5 video Demonstration of the impulse drill mechanism (cf. Fig 6). (MP4)

S6 video Demonstration of magnetically actuated needle (cf. Fig 7). (MP4)

S7 video Controlled levitation of a superconductor by steering the field-free point (cf. Fig 8). (MP4)

S8 video Reconstructed MPI data showing 2D motion of the sample (cf. Fig 9C). (MP4)
